# Supplementary material for: Frontal Transcranial Direct Current Stimulation in Moderate to Severe Depression: Clinical and Neurophysiological Findings from a Pilot Study
Source: Brain Sci. 2025 May 22;15(6):540. doi: 10.3390/brainsci15060540 (PMC12191196; doi:10.3390/brainsci15060540)
Supplement: Supplementary file 1 [file brainsci-15-00540-s001.zip › brainsci-3649588-supplementary.pdf]

Supplementary Material

Frontal transcranial direct current stimulation in moderate to severe depression: Clinical and neurophysiological findings from a pilot study

Florin Zamfirache[1,#], Gabriela Prundaru[1,#], Cristina Dumitru[2,@], Beatrice Mihaela Radu[1]

Table S1. MADRS scores at baseline (MADRS_Start) and post-treatment (MADRS_End) for individual participants with **moderate to severe depression**, along with their corresponding delta values (Delta_MADRS), percentage change (MADRS_Percent_Change), and clinical response (Responder). A clinical response is defined as a ≥50% reduction in MADRS score. Participants with a percentage change in MADRS score greater than or equal to 50% are marked as "TRUE" responders, while those with less than a 50% reduction are marked as "FALSE."

Patient	MADRS_ Start	MADRS_ End	Delta_MA DRS	MADRS_ Percent_Change	Responder
A.P	36	11	-25	-69.4444	TRUE
C.P.	36	3	-33	-91.6667	TRUE
D.G.	32	18	-14	-43.75	FALSE
C.M.	29	35	6	20.68966	FALSE
M.G.	29	16	-13	-44.8276	FALSE
R.T.	27	13	-14	-51.8519	TRUE
S.B.	26	13	-13	-50	TRUE
D.O.	22	9	-13	-59.0909	TRUE
E.G.	22	10	-12	-54.5455	TRUE
R.L.	20	5	-15	-75	TRUE
È˜.M.	20	17	-3	-15	FALSE
A.F.P.	20	6	-14	-70	TRUE
R.C.	20	5	-15	-75	TRUE
A.R.	20	4	-16	-80	TRUE

Table S2. MADRS scores at baseline (MADRS_Start) and post-treatment (MADRS_End) for individual participants with **mild depression**, along with their corresponding delta values (Delta_MADRS), percentage change (MADRS_Percent_Change), and clinical response (Responder). A clinical response is defined as a ≥50% reduction in MADRS score. Participants with a percentage change in MADRS score greater than or equal to 50% are marked as "TRUE" responders, while those with less than a 50% reduction are marked as "FALSE.

Patient	MADRS_ Start	MADRS_ End	Delta_ MADRS	MADRS_ Percent_Change	Responder
D.T.	19	20	1	5.263158	FALSE
L.D.	19	12	-7	-36.8421	FALSE
V.L.	18	16	-2	-11.1111	FALSE
O.D.	18	10	-8	-44.4444	FALSE
A.A.	16	8	-8	-50	TRUE
G.P.	16	8	-8	-50	TRUE
A.I.	16	9	-7	-43.75	FALSE
M.B.	13	12	-1	-7.69231	FALSE
E.G.M.	12	8	-4	-33.3333	FALSE
O.R.	12	8	-4	-33.3333	FALSE
E.C.	11	8	-3	-27.2727	FALSE
G.D.	15	10	-5	-33.3333	FALSE
M.S.	10	10	0	0	FALSE
S.A.L.	11	9	-2	-18.1818	FALSE
E.B.	8	3	-5	-62.5	TRUE

Table S3. FAA results for the moderate/severe depression group before (FAA_Start) and after tDCS treatment (FAA_End), along with the change in FAA (FAA_Change). Positive changes in FAA indicate increased left frontal activity, while negative changes suggest a decrease in left frontal activation. The table presents individual patient data showing the baseline and post-treatment FAA values and their corresponding changes.

Patient	FAA_Start	FAA_End	FAA_Change
A.P	0.96	0.95	-0.01
C.P.	1.17	1	-0.17
D.G.	0.73	1.33	0.6
M.G.	0.77	1.03	0.26
R.T.	0.9	1.05	0.15
S.B.	1.05	1.34	0.29
D.O.	1.18	0.89	-0.29
E.G.	1.14	0.87	-0.27
R.L.	0.87	0.82	-0.05
È˜.M.	0.87	0.82	-0.05
A.F.P.	0.9	1.05	0.15
R.C.	0.92	1.03	0.11
A.R.	0.77	1.03	0.26
E.G.	0.9	1.05	0.15

Table S4. FAA results for the mild depression group before (FAA_Start) and after tDCS treatment (FAA_End), along with the change in FAA (FAA_Change). Positive changes in FAA indicate increased left frontal activity, while negative changes suggest a decrease in left frontal activation. The table presents individual patient data showing the baseline and post-treatment FAA values and their corresponding changes.

Patient	FAA_Start	FAA_End	FAA_Change
O.D.	0.95	0.9	-0.05
G.P.	0.85	0.85	0
A.A.	0.77	0.77	0
E.C.	0.9	0.95	0.05
G.D.	1.2	1.2	0
M.B.	0.85	0.85	0
M.D.	1.08	1.08	0

Table S5. Theta/Alpha ratios at F3 and F4 for the moderate/severe depression group before (Start) and after (End) tDCS treatment, along with the change in Theta/Alpha ratios (ThetaAlpha F3_Change and ThetaAlpha F4_Change). Positive changes in Theta/Alpha ratios reflect an increase in cortical activation, while negative changes suggest decreased activation. The table presents individual patient data for the baseline and post-treatment Theta/Alpha ratios at both F3 and F4 sites, as well as their corresponding changes.

Patient	ThetaAlpha F3_Start	ThetaAlpha F3_End	ThetaAlpha F4_Start	ThetaAlpha F4_End	ThetaAlpha F3_Change	ThetaAlpha F4_Change
A.P	1.53	1.11	1.29	1.19	-0.42	-0.1
C.P.	0.98	1.03	1.37	0.97	0.05	-0.4
D.G.	0.62	1.79	0.47	1.63	1.17	1.16
M.G.	0.93	0.93	0.72	1.56	0	0.84
R.T.	0.88	1	0.83	1.08	0.12	0.25
S.B.	0.96	0.96	1.28	1.35	0	0.07
D.O.	0.63	0.82	0.77	0.51	0.19	-0.26
E.G.	0.79	1.21	0.81	0.96	0.42	0.15
R.L.	0.79	1.21	0.96	1.44	0.42	0.48
È˜.M.	1.21	1.54	0.96	1.44	0.33	0.48
A.F.P.	0.88	1	0.83	1.08	0.12	0.25
R.C.	1	1	0.97	1.33	0	0.36
A.R.	0.93	0.93	0.72	1.56	0	0.84
E.G.	0.88	1	0.83	1.08	0.12	0.25

Table S6. Theta/Alpha ratios at F3 and F4 for the mild depression group before (Start) and after (End) tDCS treatment, along with the change in Theta/Alpha ratios (ThetaAlpha F3_Change and ThetaAlpha F4_Change). Positive changes in Theta/Alpha ratios reflect an increase in cortical activation, while negative changes suggest decreased activation. The table presents individual patient data for the baseline and post-treatment Theta/Alpha ratios at both F3 and F4 sites, as well as their corresponding changes.

Patient	ThetaAlpha F3_Start	ThetaAlpha F3_End	ThetaAlpha F4_Start	ThetaAlpha F4_End	ThetaAlpha F3_Change	ThetaAlpha F4_Change
O.D.	0.74	0.88	0.88	0.7	0.14	-0.18
G.D.	1.1	1.1	1.07	1.07	0	0
M.B.	0.79	0.74	0.81	0.81	-0.05	0
E.C.	1.1	1.1	1.07	1.07	0	0
G.P.	0.79	0.79	0.81	0.81	0	0
A.I.	1.09	1.09	1.09	1.09	0	0
M.D.	1.09	1.09	1.09	1.09	0	0

Table S7. PQ-9 Symptom Improvement Summary

Symptom	Mean Change	Std Dev	p-value	Cohen's d	Significant
Mood	1.9	2.18	0.0224	0.87	Yes
Unease	0.8	1.55	0.1369	0.52	No
Sleep	2.1	1.97	0.0082	1.07	Yes
Appetite	0.3	1.83	0.6164	0.16	No
Concentration	2	1.76	0.0059	1.13	Yes
Initiative	1.1	0.99	0.0067	1.11	Yes
Involvement	2	1.89	0.0085	1.06	Yes
Pessimism	1.6	1.65	0.0133	0.97	Yes
Zest	1.1	1.6	0.0571	0.69	No
